# Dipolophoresis and Travelling-Wave Dipolophoresis of Metal Microparticles

**DOI:** 10.3390/mi11030259

**Published:** 2020-02-28

**Authors:** Jose Eladio Flores-Mena, Pablo García-Sánchez, Antonio Ramos

**Affiliations:** 1 Facultad de Ciencias de la Electrónica, Benemérita Universidad Autónoma de Puebla, Av. San Claudio y 18 Sur, San Manuel, CU. FCE2, Puebla 72570, Mexico; jefloresmena@gmail.com; 2 Departamento de Electrónica y Electromagnetismo, Facultad de Física, Universidad de Sevilla, Avda. Reina Mercedes s/n, 41012 Sevilla, Spain; pablogarcia@us.es

**Keywords:** electrokinetics, dipolophoresis, dielectrophoresis, induced-charge electrophoresis, nanowires

## Abstract

We study theoretically and numerically the electrokinetic behavior of metal microparticles immersed in aqueous electrolytes. We consider small particles subjected to non-homogeneous ac electric fields and we describe their motion as arising from the combination of electrical forces (dielectrophoresis) and the electroosmotic flows on the particle surface (induced-charge electrophoresis). The net particle motion is known as dipolophoresis. We also study the particle motion induced by travelling electric fields. We find analytical expressions for the dielectrophoresis and induced-charge electrophoresis of metal spheres and we compare them with numerical solutions. This validates our numerical method, which we also use to study the dipolophoresis of metal cylinders.

## 1. Introduction

The precise control of small particles in liquid suspension is possible by application of AC electric fields [1,2]. In particular, manipulation of metal and semiconducting particles dispersed in aqueous electrolytes has received much attention in the last decade. Examples of particle manipulation by AC fields include the transport of metal spheres and nanowires [3,4], orientation of metal [5,6,7,8] and semiconducting nanowires [9,10,11], continuous rotation of metal spheres and nanowires [12,13,14,15,16,17], and self-assembly of metal nanowires [18,19]. Additionally, the electrical manipulation of metallo-dielectric Janus spheres has been recently investigated and electrokinetic phenomena such as transport [20,21], orientation [22], rotation [23], and assembly [24] of Janus spheres have been demonstrated.

Several works are focused on the theoretical modelling of the electrokinetic behavior of metal particles [25,26,27]. In general, these works show that the electrical response of metal microparticles is determined by the formation of an induced electrical double layer (EDL) at the particle surface—that is, at the interface between the particle and the electrolyte. The applied electric field not only induces charges within the EDL, but also interacts with them and gives rise to a series of phenomena commonly referred to as induced-charge electrokinetics [28,29]. In contrast, the electrokinetics of insulating particles is mainly determined by the intrinsic surface charge that naturally appears at solid-electrolyte interfaces [30]. This charge is only slightly perturbed by applied electric fields. Several theoretical papers deal with the electrorotation and electro-orientation of metal nanowires and elongated particles in the limit of thin EDL [8,31,32,33,34]. The electrorotation of metal and semiconducting spheres with arbitrary thickness of the EDL is theoretically described in [35,36]. The particle displacement when a metal sphere is subjected to a non-homogeneous DC field was first studied by Shilov and Simonova [37] and they coined the term dipolophoresis (DIP) to refer to this phenomenon. Miloh extended this term to the more general case of AC electric fields [25,26]. Most theoretical works identify two distinct mechanisms that can lead to motion of a metal particle [27]: (i) the electrical force acting on the induced charges, and the motion induced by this mechanism is known as dielectrophoresis (DEP); and (ii) the particle displacement generated by the induced-charge electroosmostic (ICEO) flows on its surface, which we refer to as induced-charge electrophoresis (ICEP) [38]. Consequently, DIP can be described as the combined effect of DEP and ICEP.

In this work, we study the DIP motion of metal spheres and cylinders subjected to AC electric fields in two different cases. First, we consider an AC field with a non-homogeneous magnitude but a homogeneous phase. The DIP of spheres in this situation has been already studied and we reproduce previous analytical results [27]. In addition, we compare these analytical expressions with numerical results. Subsequently, we exploit the same approach to study the DIP of metal cylinders: numerical results for the DEP and ICEP of metal cylinders with different aspect ratios are reported for the first time. Secondly, we also consider an AC field with a space-dependent phase. In particular, we assume that the metal particles are subjected to travelling-wave electric fields and the motion that emerges is named as travelling-wave DIP (twDIP). Travelling-wave dipolophoresis of a polarizable colloid in a pore was considered by Miloh and Boymelgreen [39] in the case of EDL overlapping between particles and walls. Here, we consider thin double layers and the electrokinetic flow induced on a single particle. Specifically, we report novel analytical expressions for the twDIP of spheres and numerical results for metal cylinders with different aspect ratios.

## 2. Theoretical Background

Our goal is to find the velocity of a metal particle suspended in an aqueous electrolyte and subjected to an electric field. As mentioned above, the particle motion can be described as the net effect of two distinct contributions: the motion that arises from the electrical forces acting on the electrolyte-particle system and the particle displacement generated by ICEO flows on the particle surface. Therefore, the net particle velocity can be written as:

(1) $$U_{\mathrm{DIP}}=U_{\mathrm{DEP}}+U_{\mathrm{ICEP}}$$

where $U_{\mathrm{DEP}}$ is the dielectrophoretic velocity, i.e., the velocity induced by electrical forces, and $U_{\mathrm{ICEP}}$ corresponds to the induced-charge electrophoretic velocity. Since the net motion of the metal particle is named dipolophoresis, we use $U_{\mathrm{DIP}}$ to indicate the net particle velocity.

In the following, we analyze this problem in two different situations: (A) a metal particle subjected to a non-homogeneous field (Dipolophoresis); and (B) a metal particle subjected to a travelling-wave electric field (Travelling-wave Dipolophoresis).

### 2.1. Dipolophoresis of a Metal Particle

**Figure 1:**
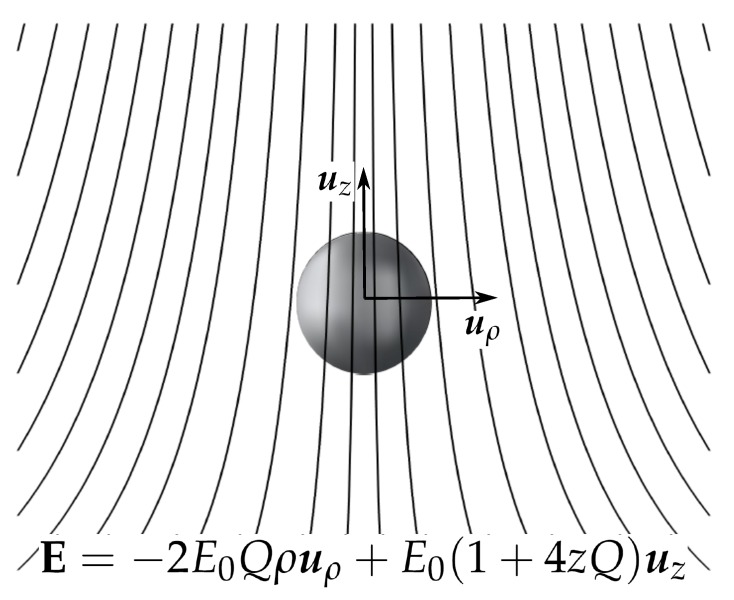
Metal particle subjected to a non-homogeneous electric field.

Let us consider a metal particle in the origin of coordinates and subjected to a non-homogeneous electrostatic field (see Figure 1). We assume that the electric field is originated by a time-harmonic electric potential given by ϕ=Re[ϕ˜expiωt], where ω is the angular frequency, Re[⋯] means the real part of [⋯], and $\tilde{\phi }$ is the electric potential phasor. We choose the following expression for the applied potential:

(2) $$\tilde{\phi }=-E_{0}\left(z-Q\left(\rho^{2}-2z^{2}\right)\right)$$

where $E_{0}$ and *Q* are constants and ρ and *z* are the cylindrical coordinates. Thus, the applied electric field has axial symmetry and can be written as:

(3) $$E=-2E_{0}Q\rho u_{\rho }+E_{0}\left(1+4zQ\right)u_{z}$$

We choose this functional form of the electric field for convenience, as shown below. We anticipate that the results that we obtain are valid for any divergent axisymmetric field as long as the particle size is smaller than the typical length scale for variation of the applied field. The electrical current driven by this applied field induces an EDL at the electrolyte–particle interface. The thickness of the EDL is given by the Debye length [30] and for aqueous electrolytes is typically around tens of nanometers or less. Therefore, the thin EDL approximation remains valid for particles with typical size in the micrometer range. We assume that the metal particle is uncharged and perfectly polarizable (i.e., there are no Faradaic currents at the metal–electrolyte interface). This assumption remains valid if the voltage drop across the EDL remains relatively small. More rigorously, as long as this voltage drop is of the order or smaller than the thermal voltage $k_{B}T/e=25$ mV, the charging of a thin EDL can be described as the charging of a capacitor with surface capacitance $C_{DL}$ [40]. Since this capacitor is charged by the current flowing in an electrolyte with conductivity σ, the following boundary condition applies for the electric potential on the particle surface *S*:

(4) $${\left.\sigma \frac{\partial \tilde{\phi }}{\partial n}\right|}_{S}=i\omega C_{DL}\left(\tilde{\phi }-V\right)$$

where ∂/∂n is the partial derivative in the direction normal to the particle surface. *V* is the potential of the metal particle and, in principle, it is determined by imposing that the total current on the particle must vanish:

(5) $$\int_{S}{\left.\frac{\partial \phi }{\partial n}\right|}_{S}dS=0$$

From the solution of the electric potential, the time-averaged electrical force on the metal particle can be computed as the flux of Maxwell stress tensor on the particle surface:

(6) $$F_{\mathrm{DEP}}=\left(1/2\right)\varepsilon \int_{S}Re\left[EE^{*}-\left(1/2\right)I\left(E\cdot E^{*}\right)\right]\cdot dS$$

where ${}^{*}$ indicates the complex conjugate, ε is the dielectric constant of the electrolyte, and I is the identity matrix.

When the particle is much smaller than the length scale for variation of the electric field, the net electrical force on the particle can be calculated as the time-averaged of the force on the induced dipole: FDEP=(1/2)Re[p˜·∇E˜*], where $\tilde{p}$ is the induced dipole phasor. For the electric field given by Equation (3), this approximation holds as long as $a\ll Q^{-1}$, where *a* is the typical size of the metal particle (*a* is the radius for the case of a sphere; for a cylinder, *a* is half of its length).

For example, in the case of a spherical particle, the induced dipole is usually written as $\tilde{p}=\tilde{\alpha }\tilde{E}$, where $\tilde{\alpha }$ is the particle polarizability. Thus, the DEP force for a spherical particle in the origin of coordinates and subjected to the field given by Equation (3) is written as:

(7) FDEP=(1/2)Re[α˜(E˜·∇)E˜*]=2Re[α˜]QE02uz

The DEP velocity ($U_{\mathrm{DEP}}$) is determined by the balance of the viscous drag on the particle and the electrical force. For example, the viscous drag on a sphere that moves with velocity v within a fluid with viscosity η is given by Stokes equation, $F_{drag}=-6\pi \eta av$. Thus, the DEP velocity is obtained as $U_{\mathrm{DEP}}=F_{\mathrm{DEP}}/6\pi \eta a$.

The induced-charge electrophoretic velocity ($U_{\mathrm{ICEP}}$) is originated by the electroosmotic slip velocity ($v_{s}$) induced at the particle surface. According to Helmholtz–Smoluchowski formula [30], and under the thin EDL approximation, the time-averaged of $v_{s}$ can be computed as [40,41]:

(8) $$v_{s}=-\left(\varepsilon /4\eta \right)\nabla_{t}{\left|\tilde{\phi }-V\right|}^{2}$$

where $\nabla_{t}$ is the gradient operator tangential to the particle surface.

Making use of the Lorentz reciprocity theorem [25,42,43], and taking the *Z*-axis as the direction of ICEP motion, i.e., $U_{\mathrm{ICEP}}=U_{\mathrm{ICEP}}u_{z}$:

(9) $$U_{\mathrm{ICEP}}=-\frac{\int_{S}\left(n\cdot T\cdot v_{s}\right)dS}{\int_{S}\left(n\cdot T\cdot u_{z}\right)dS}$$

where T is the hydrodynamic stress tensor that arises when the particle displaces with a certain velocity. Thus, the denominator in Equation (9) corresponds to the viscous drag on the particle.

Equation (9) allows us to compute the particle ICEP velocity without solving the fluid velocity field (Stokes problem) induced by the slip velocity. Instead, we only need to know the stress tensor associated to the velocity field induced by a particle that displaces within the fluid.

### 2.2. Travelling-Wave Dipolophoresis

**Figure 2:**
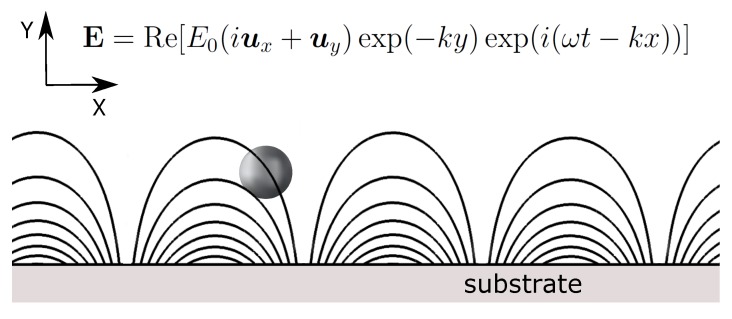
Metal particle subjected to a travelling electric field.

In the previous section, we consider the motion of a metal particle subjected to an AC electric field with a non-homogeneous magnitude but a homogeneous phase (Equation (3)). This is not always the case and, for instance, several applications are based on the use of travelling-wave electric fields that induce a continuous displacement of particles [44]. For example, let us consider the electric field in Figure 2. This field is obtained when the substrate (y=0) is subjected to a travelling-wave electric potential of the form V=V0cos(ωt−kx)=Re[V0exp(i(ωt−kx))], where $V_{0}$ is the amplitude of the applied voltage and *k* is the wavenumber. Thus, $E_{0}=kV_{0}$ in the expression of Figure 2. The electric force that appears with this field has two components: (i) a vertical component due to the spatial dependence of the magnitude of the electric field with the y-coordinate; and (ii) a horizontal component that arises from the dependence of the phase on the x-coordinate. In effect, the electrical force can be calculated as the force on an induced dipole for particles smaller than $k^{-1}$:

(10) FDEP=(1/2)Re[α˜(E˜·∇)E˜*]=−kE02exp(−2ky)(Im[α˜]ux+Re[α˜]uy)

The x-component of the force in Equation (10) is commonly known as travelling-wave DEP force [45], while the y-component is the “conventional” DEP force, as in the previous section.

Besides the electrical force on the particle, ICEO flows induced by travelling-wave fields also affect the net particle motion. In general, the effect of a travelling-wave field on a metal particle can be described as the superposition of two distinct mechanisms: the velocity induced by the net electrical forces on the particle (commonly known as travelling-wave dielectrophoresis [45]) and the particle velocity due to the induced electroosmotic flows on the particle surface. By analogy with the previous case, we name the particle motion induced by these flows as twICEP. Likewise, we name the net motion of the metal particle subjected to travelling wave fields as travelling-wave dipolophoresis (twDIP). Accordingly, we write the net particle velocity

(11) $$U_{\mathrm{twDIP}}=U_{\mathrm{twDEP}}+U_{\mathrm{twICEP}}$$

As in the previous section, $U_{\mathrm{twDEP}}$ is calculated from the balance between the time-averaged electrical force and the viscous drag, while $U_{\mathrm{twICEP}}$ is calculated by using the reciprocity theorem in Equation (9).

## 3. Mathematical Methods and Results

Our goal is to find the dipolophoretic and travelling-wave dipolophoretic velocities for spherical microparticles and for nanowires. We first consider spheres because we can find analytical solutions for the particle velocity. This allows us to check our numerical methods against analytical results. Later, the dipolophoretic motion of a metal cylinder is numerically studied.

### 3.1. Dipolophoresis of a Metal Sphere

Let us consider a metal sphere with radius *a* in the origin of coordinates and subjected to a harmonic non-homogeneous electric field, as in Section (Figure 1). We look for the solution of Laplace equation for the electric potential phasor ($\nabla^{2}\tilde{\phi }=0$) subjected to boundary condition in Equation (4) on the sphere surface. Far from the particle, we impose that $\nabla \tilde{\phi }$ is given by the applied electric field phasor (Equation (3)). This choice of applied electric field ensures that integration of the current density is zero on any surface enclosing the particle and, in particular, on the sphere surface. Therefore, Equation (5) is satisfied. In this case, the potential of the metal particle is an arbitrary constant and we choose V=0. Thus, the boundary condition on the particle surface (r=a) is $\partial \tilde{\phi }{/\partial r|}_{a}=i\left(\omega C_{DL}/\sigma \right)\tilde{\phi }$.

From linearity of Laplace equation, the solution to the electric potential can be written as the superposition of two solutions: the potential associated to the homogeneous applied field ($E_{0}a{\tilde{\phi }}_{1}$) and the potential associated to the non-homogeneous part of the applied field (that can be written as $E_{0}a^{2}Q{\tilde{\phi }}_{2}$). Thus, we write $\tilde{\phi }=E_{0}a\left({\tilde{\phi }}_{1}+\bar{Q}{\tilde{\phi }}_{2}\right)$, where $\bar{Q}=aQ$ is a non-dimensional parameter that characterizes the non-uniformity of the applied field. Taking *a* as the scale for distances, boundary conditions far from the particle are $\bar{\nabla }{\tilde{\phi }}_{1}=-u_{z}$ and $\bar{\nabla }{\tilde{\phi }}_{2}=\left(-4\bar{z}u_{z}+2\bar{\rho }u_{\rho }\right)$, where $\bar{\nabla }=a\nabla$, $\bar{z}=z/a$ and $\bar{\rho }=\rho /a$. In addition, after defining a non-dimensional angular frequency as $\Omega =\omega C_{DL}a/\sigma$, boundary conditions on the sphere surface are $\partial {\tilde{\phi }}_{1}/\partial \bar{r}{|}_{S}=i\Omega {\tilde{\phi }}_{1}$ and $\partial {\tilde{\phi }}_{2}/\partial \bar{r}{|}_{S}=i\Omega {\tilde{\phi }}_{2}$, ($\bar{r}=r/a$).

The solutions for ${\tilde{\phi }}_{1}$ and ${\tilde{\phi }}_{2}$ in spherical coordinates can be written as follows:

(12) $$\begin{array}{ccc} {\tilde{\phi }}_{1} & = & -\bar{r}\cos\left(\theta \right)+\frac{A_{1}}{{\bar{r}}^{2}}\cos\left(\theta \right) \end{array}$$

(13) $$\begin{array}{ccc} {\tilde{\phi }}_{2} & = & -2{\bar{r}}^{2}P_{2}\left[\cos\left(\theta \right)\right]+\frac{A_{2}}{{\bar{r}}^{3}}P_{2}\left[\cos\left(\theta \right)\right] \end{array}$$

where $P_{2}\left[x\right]$ indicates the Legendre polynomial of order 2 ($P_{2}\left[x\right]=\left(1/2\right)\left(3x^{2}-1\right)$.). The constants $A_{1}$ and $A_{2}$ are determined from the boundary conditions:

(14) $$\begin{array}{ccc} A_{1} & = & \frac{-1+i\Omega }{2+i\Omega } \end{array}$$

(15) $$\begin{array}{ccc} A_{2} & = & 2\frac{-2+i\Omega }{3+i\Omega } \end{array}$$

From the solution of the electric potential phasor, the DEP force can be found by integration of Maxwell stress tensor (Equation (6)):

(16) FDEP=8πεa2Re[A1]Q¯E02uz

Alternatively, the DEP force can be found from the particle polarizability. Considering the solution of the electric potential when the applied filed is homogeneous ${\tilde{\phi }}_{1}$, the dipole term allows us to identify $\tilde{\alpha }=4\pi \varepsilon a^{3}A_{1}$. Using Equation (7), the DEP force is FDEP=8πεa2Re[A1]Q¯E02uz, as found from Equation (6). This means that multipoles of order higher than two do not contribute to the net force on the metal sphere, as expected from the symmetry of the applied field.

The DEP velocity results from the balance of the DEP force and the viscous drag:

(17) $$U_{\mathrm{DEP}}=\frac{8\varepsilon \bar{Q}E_{0}^{2}a}{6\eta }\left(\frac{-2+\Omega^{2}}{4+\Omega^{2}}\right)$$

The electroosmotic slip velocity can also be calculated from the solution of the electric potential by applying Equation (8), $v_{s}=-\left(\varepsilon /4\eta \right)E_{0}^{2}a^{2}\nabla_{t}\left({\tilde{\phi }}_{1}{\tilde{\phi }}_{1}^{*}+\bar{Q}{\tilde{\phi }}_{1}{\tilde{\phi }}_{2}^{*}+\bar{Q}{\tilde{\phi }}_{2}{\tilde{\phi }}_{1}^{*}+{\bar{Q}}^{2}{\tilde{\phi }}_{2}{\tilde{\phi }}_{2}^{*}\right)$. The terms ${\tilde{\phi }}_{1}{\tilde{\phi }}_{1}^{*}$ and ${\tilde{\phi }}_{2}{\tilde{\phi }}_{2}^{*}$ give rise to slip velocity fields that are symmetric with respect to the plane z = 0 and, therefore, they do not contribute to the particle ICEP velocity. Thus, we only have to consider the slip velocity induced by the cross-terms ${\tilde{\phi }}_{1}{\tilde{\phi }}_{2}^{*}$ and ${\tilde{\phi }}_{2}{\tilde{\phi }}_{1}^{*}$:

(18) $$\begin{array}{ccc} v_{s}\left(\mathrm{cross}-\mathrm{terms}\right) & = & -\frac{\varepsilon \bar{Q}E_{0}^{2}a}{4\eta }\frac{\partial }{\partial \theta }\left({\tilde{\phi }}_{1}{\tilde{\phi }}_{2}^{*}+{\tilde{\phi }}_{2}{\tilde{\phi }}_{1}^{*}\right)u_{\theta }=\cdots \end{array}$$

(19) ⋯=εQ¯E02a8ηsin(θ)(7+9cos(2θ))ReA1−1A2*−2uθ

where the gradient operator tangential to the sphere surface is written as $\nabla_{t}=\left(1/a\right)\left(\partial /\partial \theta \right)u_{\theta }$. Application of the Lorentz reciprocity theorem (Equation (9)) for a spherical particle leads to the following integral for $U_{\mathrm{ICEP}}$ [38]: $U_{\mathrm{ICEP}}u_{z}=-\left(1/2\right)\int v_{s}\sin\left(\theta \right)d\theta$. The ICEP velocity of the metal sphere is:

(20) $$U_{\mathrm{ICEP}}=\frac{4a\varepsilon \bar{Q}E_{0}^{2}}{\eta }\left(\frac{6+\Omega^{2}}{36+13\Omega^{2}+\Omega^{4}}\right)$$

More generally, the DEP and ICEP velocities of a metal sphere can be written as:

(21) $$\begin{array}{ccc} U_{\mathrm{DEP}} & = & \frac{\varepsilon a^{2}}{6\eta }\left(\frac{-2+\Omega^{2}}{4+\Omega^{2}}\right)\nabla E^{2} \end{array}$$

(22) $$\begin{array}{ccc} U_{\mathrm{ICEP}} & = & \frac{a^{2}\varepsilon }{2\eta }\left(\frac{6+\Omega^{2}}{36+13\Omega^{2}+\Omega^{4}}\right)\nabla E^{2} \end{array}$$

where we take into account that $\nabla E_{0}^{2}=8\left(\bar{Q}E_{0}^{2}/a\right)u_{z}$ at the origin of coordinates. These results were already obtained in [27]. For DC (Ω=0), they are coincident with those found in [37].

### 3.2. Travelling-Wave Dipolophoresis of a Metal Sphere

Our goal in this section is to study the motion of a metal particle subjected to an AC field with a non-homogeneous phase. To this end, we consider that a sinusoidal travelling wave potential is applied to the wall of an infinite cylinder of radius *R*. If the cylinder axis coincides with the Z-axis of a cylindrical system of coordinates, the applied potential can be written as $V=V_{0}\exp\left[i\left(\omega t-kz\right)\right]$, where $V_{0}$ and *k* are constants. Thus, from the solution of Laplace equation, the electric potential inside the cylinder in cylindrical coordinates is $\phi \left(r,z\right)=V_{0}\exp\left[i\left(\omega t-kz\right)\right]I_{0}\left(k\rho \right)$, where $I_{0}\left(x\right)$ is the modified Bessel function of first order. We assume that a metal particle is at position ρ=0,z=0. If the particle size is much smaller than *R* and $k^{-1}$, the electric potential phasor in the particle proximity can be written as (keeping terms up to second order): $\tilde{\phi }\left(\rho ,t\right)\approx V_{0}\left(1-ikz+k^{2}/4\left(\rho^{2}-z^{2}\right)\right)$. Thus, we consider that the particle is subjected to an electric field with a phasor given by $\tilde{E}\left(\rho ,t\right)=V_{0}\nabla \left(ikz-\left(k^{2}/4\right)\left(\rho^{2}-z^{2}\right)\right)$ or, in spherical coordinates:

(23) $$\tilde{E}\left(r,t\right)=ikV_{0}\nabla \left(r\cos\left(\theta \right)-\left(ik/2\right)r^{2}P_{2}\left[\cos\left(\theta \right)\right]\right)$$

This expression is equivalent to Equation (3) after identifying $E_{0}=ikV_{0}$ and Q=−ik/4. The force on the induced dipole is:

(24) FtwDEP=(1/2)Re[α˜(E˜·∇)E˜*]=8πεa2E02Re[A1ik/4]uz=2πεk¯3V02Im[A]uz

where $\bar{k}=ka$. This result corresponds to a pure travelling-wave DEP force. Using Equation (14) and balancing electrical and viscous forces on the spheres, the travelling-wave DEP velocity is:

(25) $$U_{\mathrm{twDEP}}=-\frac{\varepsilon \bar{k}aE_{0}^{2}}{\eta }\frac{\Omega }{4+\Omega^{2}}$$

the negative sign indicates that the directions of the DEP velocity and the travelling-wave propagation are opposite.

The ICEP velocity induced by the travelling-wave is determined by the cross-terms in the expression for the slip velocity:

(26) vs(cross−terms)=−iεkE02a216η∇t(ϕ1ϕ2*−ϕ2ϕ1*)=εkE02a28ηIm[∇t(ϕ1ϕ2*)]

As before, we make use of the reciprocity theorem and, using Equations (14) and (15), the travelling-wave ICEP velocity results:

(27) $$U_{\mathrm{twICEP}}=\frac{\varepsilon \bar{k}aE_{0}^{2}}{\eta }\frac{\Omega }{\left(4+\Omega^{2}\right)\left(9+\Omega^{2}\right)}$$

Hence, the ICEP contribution is in the same direction of the travelling-wave propagation, although smaller than the DEP motion by a factor $\left(9+\Omega^{2}\right)$.

### 3.3. Comparison with Numerical Results for Spheres

In this section, we show how to calculate numerically the dipolophoretic motion of metal spheres in the same situations of Section 3.1 and Section 3.2. Thus, we can check the results of the numerical method against the analytical expressions above.

**Figure 3:**
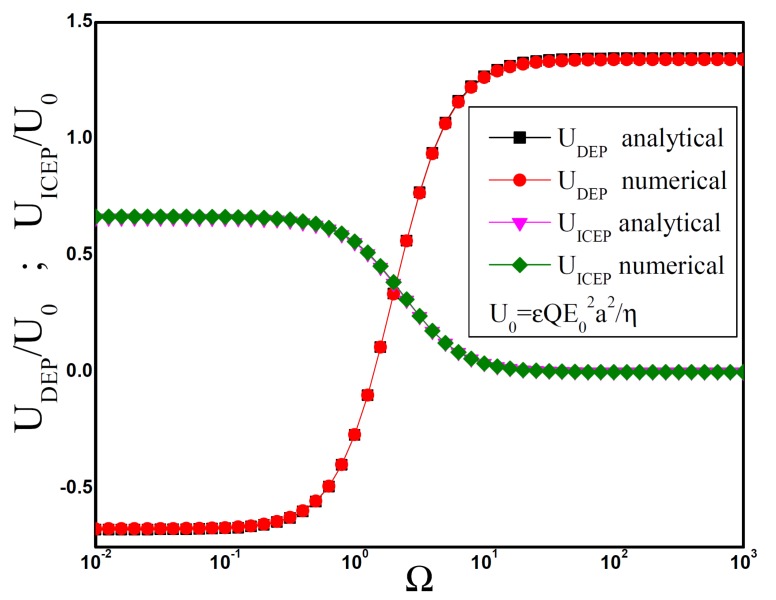
Dipolophoresis of a metal sphere as a function of angular frequency of the applied ac field. Analytical and numerical results are compared. The velocity is non-dimensionalized with $U_{0}=\varepsilon QE_{0}^{2}a^{2}/\eta$.

We used the software Comsol Multiphysics to calculate the electric potential in the electrolyte. Since the problem has axial symmetry, we solve the Laplace equation in a square domain whose side is ten times longer than the sphere radius. The boundary condition on the sphere surface is Equation (4) with V=0. We impose that the electric field far from the spheres is given by Equation (3), with $E_{0}=1$ and $\bar{Q}=0.1$. From the solution of the electric potential, we calculated the DEP force on the sphere by using the Maxwell stress tensor in Equation (6) and from the sphere polarizability. As in the previous section, the DEP velocity is obtained by balancing the DEP force with the viscous drag. Figure 3 shows the numerical results for these two methods. It also shows the analytical solution in the section above.

Maximum DEP velocities are obtained for Ω>1. For a KCl water solution with concentration $10^{-4}$ M, the liquid conductivity is σ=1.5 mS/m and, according to the Debye–Huckel formula, the EDL specific capacitance is $C_{DL}\approx 0.023$ $F/m^{2}$. Thus, for a particle with radius a=5μm, the maximum DEP velocity is obtained for f=ω/2π>2 kHz. An estimate of this velocity can be obtained if we use some typical values for the applied electric field in experiments, $E_{0}^{2}=10^{8}$ ${\left(V/m\right)}^{2}$, $\bar{Q}=0.1$. Maximum $U_{DEP}$ is around 50 μm/s.

We also calculated numerically the ICEP velocity of a sphere by applying Equation (9), where T is the viscous stress tensor that arises from the flow field induced by the motion of a sphere with a given velocity. In principle, this flow field could be found by considering that the fluid velocity is zero far from the particle, but an accurate numerical solution of this problem requires a domain much larger than, for example, the domain we used for the electrical problem. However, we can greatly reduce the size of the domain if we make use of the well-known solution for the fluid velocity and pressure generated by a point force of magnitude *F* [46], which in cylindrical coordinates reads:

(28) $$v_{\rho }=\frac{F}{8\pi \eta }\frac{\rho z}{{\left(\rho^{2}+z^{2}\right)}^{3/2}};\;v_{z}=\frac{F}{8\pi \eta }\frac{\rho^{2}+2z^{2}}{{\left(\rho^{2}+z^{2}\right)}^{3/2}};\;p=\frac{F}{4\pi }\frac{z}{{\left(\rho^{2}+z^{2}\right)}^{3/2}}$$

Thus, we apply the boundary conditions in Equation (28) on the boundaries far from the particle. The problem is closed by imposing that the parameter *F* is equal to the viscous drag on the particle:

(29) $$F=\int_{S}n\cdot T\cdot u_{z}dS$$

where *S* is the particle surface. We obtained F/ηa=18.7905, a relative difference of 0.6% with respect to the Stokes law.

From the solution of the Stokes problem, we obtained $U_{\mathrm{ICEP}}$ by numerical integration of Equation (9) on the sphere surface. Figure 3 shows the numerical results together the analytical results obtained in the previous section. Maximum ICEP velocities are obtained for Ω<1. We can estimate this velocity for the same experimental parameters that we considered in the estimation of maximum $U_{DEP}$. Thus, maximum $U_{ICEP}$ in these conditions is around 25 μm/s and it is obtained when f=ω/2π<2 kHz.

**Figure 4:**
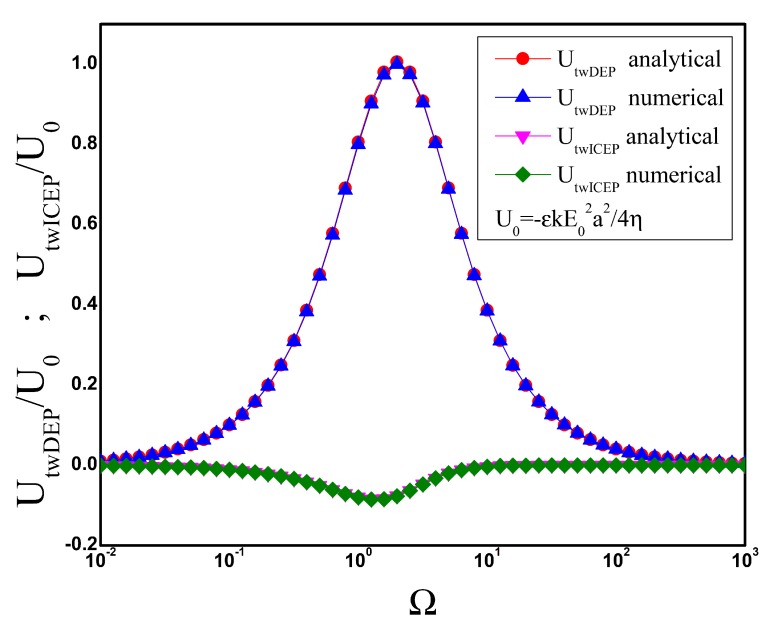
Travelling-wave dipolophoresis of a metal sphere as a function of angular frequency of the applied ac field. Analytical and numerical results are compared. The velocity is non-dimensionalized with $U_{0}=-\varepsilon kE_{0}^{2}a^{2}/4\eta$.

Likewise, $U_{\mathrm{twDEP}}$ and $U_{\mathrm{twICEP}}$ are calculated from the numerical solution of the electric potential when the electric field far from the particle is given by Equation (23). Figure 4 is a plot of the numerical together the analytical results obtained in the previous section. Both twDEP and twICEP arise from the interaction of the electric field with the out-of-phase charge within the EDL and, thus, the time-averaged velocity vanishes at low and high frequencies.

### 3.4. Dipolophoresis and Travelling-Wave Dipolophoresis of a Metal Cylinder

Our goal in this section is to calculate the dipolophoresis and travelling-wave dipolophoresis of a metal nanowire by means of the same numerical procedure that we used for the metal spheres. Thus, we start by assuming that we have a metal cylinder at the origin of coordinates and subjected to an electric field given by Equation (3). The aspect ratio of the metal cylinder is β=b/a, where *b* is the cylinder radius and 2a is the cylinder length. We also assume that the long axis of the metal cylinder is aligned with the Z-axis. We choose this orientation because it is well known, both experimentally and theoretically [7,34], that a metal nanowire orients parallel to the electric field for any value of the applied frequency. With this orientation, the problem is 2D axisymmetric and we use the same computational domain as for the spheres. Obviously, we use the same boundary condition for the electric potential on the surface of the metal.

**Figure 5:**
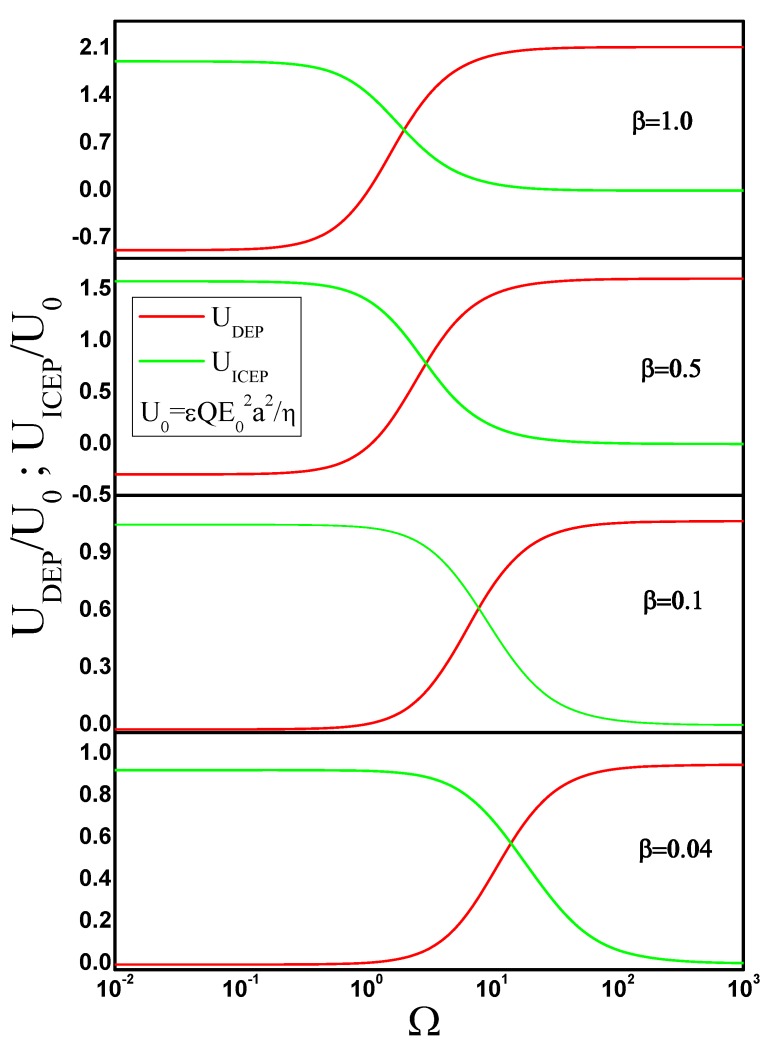
Dipolophoresis of a metal cylinder as a function of angular frequency of the applied ac field. Results are shown for four different values of the cylinder aspect ratio β=b/a. The velocity is non-dimensionalized with $U_{0}=\varepsilon QE_{0}^{2}a^{2}/\eta$.

From the solution of the electric potential, the DEP force on the metal nanowire is found from the integration of the time-averaged Maxwell stress tensor. The DEP velocity of the nanowire is found by balancing the electrical force with the viscous drag, which can be written as $F_{\mathrm{viscous}}=\gamma v$, where γ is the drag coefficient and v is the nanowire velocity. We numerically computed γ for a cylinder moving in the direction of its axis. To this end, we used the same approach as in the previous section and solved the Stokes equations in a domain that is only ten times larger than the cylinder length. The boundary conditions in Equation (28) are imposed far from the cylinder and the constraint in Equation (29) is evaluated on the cylinder surface. Thus, γ=F if the cylinder velocity is 1. The results for the DEP velocity are shown in Figure 5 for several values of the cylinder aspect ratio. We include β=0.04 because this is the aspect ratio of silver nanowires in [13,14]. These nanowires are candidates for experimental verification of our numerical results.

We numerically obtained γ/aη=4.1045 for a cylinder with aspect ratio β=0.04. It interesting to compare this result with the analytical expression in [47] for the viscous friction of slender cylinders, γ=4πηa/(δ−Ln(β)), where $\delta =-0.207+0.90\beta -0.133\beta^{2}$. The difference between the two values is around 0.35%.

We also obtained the DEP force from the expression of the time-averaged electrical force on the induced dipole. For a cylindrical particle, the Z component of this force can be written as Fz=(1/2)Re[(α˜‖E˜‖+α˜⊥E˜⊥)·∇)E˜z*], where ${\tilde{\alpha }}_{\|}$ and ${\tilde{E}}_{\|}$ indicate, respectively, the particle polarizability and the component of the electric field parallel to the cylinder axis. Likewise, ${\tilde{\alpha }}_{⊥}$ and ${\tilde{E}}_{⊥}$ are the particle polarizability and the component of the electric field perpendicular to the cylinder axis. As mentioned above, we consider that the metal cylinder subjected to the field given by Equation (3) is at the origin of coordinates and aligned with the Z-axis, $\tilde{\alpha_{\|}}={\tilde{\alpha }}_{z}$. Thus, ${\tilde{E}}_{⊥}=0$ and the force is:

(30) Fz=(1/2)Re[(α˜zE˜z·∇)E˜z*]

As in the case of a sphere, the cylinder polarizability can be obtained from the solution of the electric potential. In this case, the solution when the applied field is homogeneous can be written as $\tilde{\phi_{1}}=-\bar{r}\cos\left(\theta \right)+\sum_{l=1}^{\infty }A_{l}P_{l}\left[\cos\left(\theta \right)\right]/{\bar{r}}^{l+1}$, where $P_{l}\left[x\right]$ is the Legendre polynomial of order *l*. Identifying $A_{1}=A={\tilde{\alpha }}_{z}/4\pi \varepsilon a^{3}$, and using the orthogonality of Legendre polynomials, we numerically calculated *A* from the following integration:

(31) $$A=\frac{3}{4}\int_{S}\left({\tilde{\phi }}_{1}+\bar{z}\right)P_{1}\left[\cos\left(\theta \right)\right]dS$$

where *S* is any spherical surface that encloses the particle. We also plot in Figure 5 the predictions for the cylinder DEP velocity calculated with Equation (7) along with the results obtained obtained from the integration of Maxwell stresses. Both results are coincident for all aspect ratios, as expected.

Since we already know the flow field generated by a cylinder moving in a direction parallel to its axis, the ICEP velocity is calculated from Equation (9), as in the case of the sphere. Figure 5 also shows $U_{\mathrm{ICEP}}$ as a function of frequency for several values of the cylinder aspect ratio.

**Figure 6:**
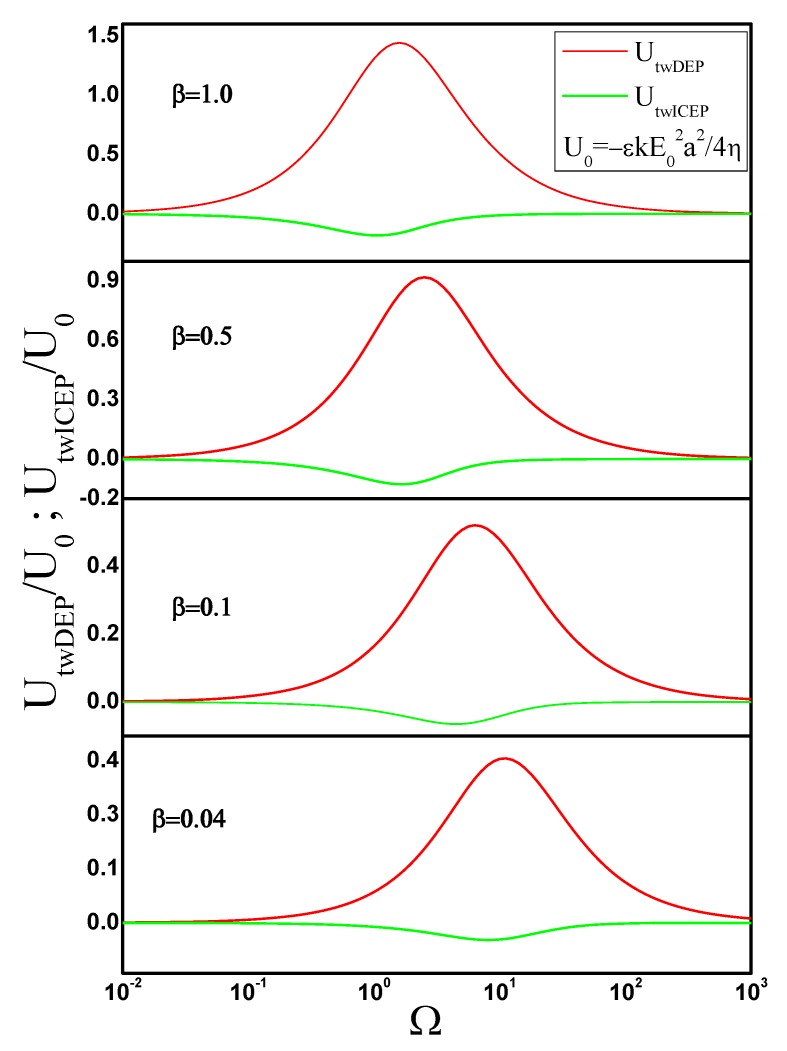
Travelling-wave dipolophoresis of a metal cylinder as a function of angular frequency of the applied ac field. Results are shown for four different values of the cylinder aspect ratio β=b/a. The velocity is non-dimensionalized with $U_{0}=-\varepsilon kE_{0}^{2}a^{2}/4\eta$.

Using the imaginary part of the cylinder polarizability, we evaluate Equation (24) to obtain the travelling-wave DEP force and, from this, the travelling-wave DEP velocity. Figure 6 shows $U_{\mathrm{twDEP}}$ as a function of frequency for several cylinder aspect ratios. We also calculated the electrical force from the expression of the Maxwell stress tensor and plot the corresponding predictions for $U_{\mathrm{twDEP}}$ in Figure 6. Both methods show perfect agreement, as expected.

Finally, the travelling-wave ICEP motion of a metal cylinder was calculated from Equation (9) and using the solution of the flow field for a moving cylinder. Figure 6 shows the results for $U_{\mathrm{twICEP}}$ as a function of angular frequency for several aspect ratios.

## 4. Conclusions

The dipolophoretic motion of metal particles subjected to ac fields is described as the combination of two distinct contributions: dielectrophoresis and induced-charge electrophoresis. The former arises from the time-averaged electrical force on the particle, while the latter is a consequence of the induced-charge electroosmotic slip velocity on the particle surface. Analytical results for the dipolophoresis of metal spheres show that they experience negative DEP at low frequencies, but it is compensated by the ICEP motion and, thus, the net dipolophoretic velocity vanishes. This is in contrast with experiments with metal spheres that demonstrate that negative DEP of metal spheres dominates at low frequencies [4]. It is well-known that ICEO flows (and the consequent ICEP motion) in experiments are generally smaller than predicted by the standard theory [48], and sometimes are about one order of magnitude smaller [27]. As frequency increases, the ICEP velocity vanishes and the particle motion is only driven by DEP forces, which are positive for metal particles and in accordance with experimental data. The travelling-wave dipolophoresis of metal spheres is dominated by the twDEP contribution, which predicts particle motion in a direction opposite to the propagation of the travelling-wave field and with a maximum velocity for frequencies around the typical RC-time for charging the sphere EDL. The twICEP contribution predicts a particle velocity in the same direction of propagation of the travelling field and much smaller than the twDEP velocity.

We also used numerical methods to calculate the dipolophoresis of metal spheres and the results are in perfect agreement with the analytical expressions. Subsequently, we used the same numerical approach for the study of the dipolophoresis of metal cylinders. For low frequencies, the calculations predict negative DEP of metal cylinders. However, the ICEP contribution is positive and much larger than the DEP. Thus, positive dipolophoresis of metal cylinders is expected at low frequencies. On the other hand, the ICEP term vanishes for high frequencies and the dipolophoresis is only determined by the DEP, which is positive for metal cylinders. In conclusion, positive dipolophoresis is predicted for all frequencies.

Finally, numerical calculations show that the twDIP of metal cylinders is completely dominated by twDEP, which predicts particle transport in a direction opposite to the propagation of the travelling-wave field and with a maximum velocity for frequencies around the typical RC-time for charging the sphere EDL, as in the case of metal spheres. The twICEP of metal cylinders is in the same direction of propagation of the travelling field, but much smaller than twDEP. With respect to a possible comparison with experimental data, it is important to keep in mind that electrokinetic manipulation of microparticles are usually made with microelectrode arrays fabricated on a glass substrate. Metal microparticles are heavier than water and precipitate. Thus, the particles rest on the glass substrate and the interaction with the wall must be accounted for.
